# Acute Cardiac Toxicity of *Nerium Oleander/Indicum* Poisoning (Kaner) Poisoning

**DOI:** 10.4103/1995-705X.76803

**Published:** 2010

**Authors:** Ibraheem Khan, Chandra Kant, Anil Sanwaria, Lokesh Meena

**Affiliations:** Department of Medicine Jawarhar Lal Nehru Medical College, Ajmer - 305 001, Rajasthan, India

**Keywords:** AV block, cardiac glycosides, *Nerium oleander*

## Abstract

We present a case of oleander leaf extract poisoning manifested by vomiting, lightheadedness, and heart block. Practicing physicians should understand the potential lethal properties of oleander and its availability throughout the world.

## INTRODUCTION

Kaner (*Nerium oleander/indicum*) is an ornamental shrub or small, densely branched tree, 1 to 10 m tall in the Dogbane family Apocynaceae. Leaves are in pairs of three or whorled, very green, leathery, narrowly elliptic to linear entire. Flowers grow in clusters in terminal branches, each 2.5 to 5 cm, funnel-shaped with five lobes, fragrant, various colors from pink to red, white, peach, and yellow.[1]

The common oleander is one of most poisonous plants that have been shown to contain nondigitalis cardiac glycosides. Oleander is an idiom for plants of the *N*. oleander L, *N. indicum*, and, *Nerium odorum*, species. Common names include soland, lorier bol, rosebay, and rose laurel and kaner.[2]

The oleander is most prevalent, and alluring flowers make it a particular hazard for accidental ingestion.[2] The plant also has shown toxicologic importance for accidents when used in folk medicines, when adults unknowingly eat parts of the plant, or food that has come into contact with the plant, such as hot-dog sticks, and in homicides or suicides. Also, as our case illustrates, toxicities are not limited to temperate climates.[3]

All parts of the oleander plant contain cardiac glycosides, including the roots and the smoke produced from burning, as heat does not inactivate the glycosides. The toxic component are the two potent cardiac glycosides, oleanderin and neriine, which can be isolated from all parts of the plant, Both are very similar to the toxin of Foxglove.[4] Both have positive inotropic, negative chronotropic, and cross reactivity. This includes direct glycoside poisoning of the sodium-potassium pump of the heart and increased vagotonia. Most symptoms from oleander poisoning are cardiac and gastrointestinal in nature and appear four hours after the ingestion.[5]

We report a case of intentional oleander ingestion.

## CASE REPORT

A 21-year-old female was admitted in the emergency room with vomiting and lightheadedness 15 hours after ingestion of common oleander aqueous leaf extract (10-20 leaves). She had been advised to take the extract in order to conceive a baby.

The patient was a non-smoker and non-alcoholic. She had no drugs allergy and was mentally sound. On initial examination, the blood pressure was 122/80 mmHg with irregular pulse of 46/min. She was looking toxic due to excessive vomiting. Other general physical parameters were normal. Her chest and lungs were clear to auscultation and percussion. Cardiovascular examination revealed an irregular rhythm with soft S1and normal audible S2 over the cardiac apex.

**Figure 1:**
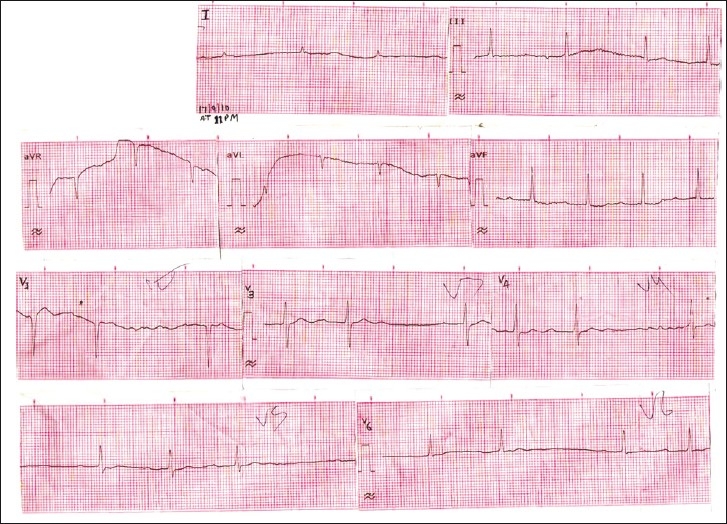
Electrocardiogram shows intermittent AV block 15 hours after the ingestion

**Figure 2:**
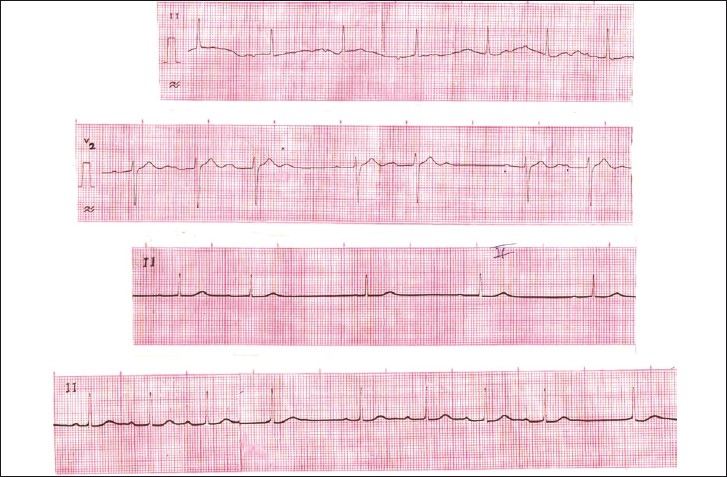
Electrocardiogram in lead II (Top) shows first degree AV block with inverted P wave and prolonged PR interval .28 sec and in lead V2 intermittent 2:1 AV block 15 hours after the ingestion. First degree AV block after .6 mg iv atropine administration in lead II (Middle). First degree AV block Type I Second-Degree AV block with atypical wenckebach periodicity with junctional rhythm after .6 mg iv atropine administration in lead II (Bottom)

Electrocardiogram revealed inverted P wave in inferior lead and prolonged PR interval (.28 s), with varying degree AV blocks and normal QRS duration [Figure 1 and 2].

The patient was given .6 mg of intravenous atropine sulfate which did not resolve her bradycardia, but other symptoms were improved.

Next day, the patient was given intravenous atropine sulfate. 6 mg twice a day and tablet orciprenaline 10 mg three times a day.

After three days, the patient was discharged on request, with sinus node dysfunction and varying degree AV blocks [Figure 2] but asymptomatic.

## DISCUSSION

Most of the plants, including foxglove and oleander, have been identified as containing cardiac glycosides and these are oleandrin, oleandroside, nerioside, digitoxigenin, thevetin, and thevetoxin.[3] The cardiac glycosides in oleander produce more gastrointestinal effects than those in digoxin, and the symptoms range from nausea and vomiting to cramping and bloody diarrhea. Also, it causes irritation to the mucosal membranes, resulting in burning around the mouth and increased salivation. Confusion, dizziness, drowsiness, weakness, visual disturbances, and mydriasis are central nervous system manifestations of toxicity.[4]

The most serious side effects of oleander poisoning are cardiac abnormalities, including various ventricular dysrhythmias, tachyarrhythmias, bradycardia, and heart block.[2] Electrocardiography often reveals an increased PR interval, a decreased QRS-T interval, and T wave flattening or inversion. It is thought that these clinical manifestations are the result of both increased vagotonia and direct cardiac glycoside toxicity.[3]

The treatment of oleander poisoning is empirically based on the treatment of digitalis-glycoside toxicity and consists of supporting the patient hemodynamically. This may include administering atropine for severe bradycardia; using phenytoin or lidocaine hydrochloride to control dysrhythmias; placing a temporary venous pacemaker; or electrical counter shock and administering digoxin-specific Fab antibody fragments (Digibind).[4]

Other treatment methods are aimed at removing the toxic substance from the stomach by emesis. Special concern must be given to a patient with bradycardia before emesis is induced because of the possibility of a vagal reaction and worsening of the bradycardia. Unabsorbed glycosides may be bound to some extent, depending on the particular glycoside, by various binding agents in the gut. These agents theoretically should be more effective in absorbing less polar glycosides, such as digitoxin, than the more polar glycosides like digoxin (for example, cholestyramine resin and colestipol). The use of these agents is not thought to have substantial value in the treatment of advanced toxicity, and they were not used in our patient.[6] Activated charcoal has been shown to be useful in preventing further absorption of the cardiac glycosides by interruption of the enterohepatic circulation of the glycoside, but it was not used in our patient because she was brought after 15 hours of ingestion of the toxin and due to the unknown status about the enterohepatic circulation of oleander’s glycosides.[6]

## CONCLUSION

It is interesting that oleander poisoning can be fatal with relatively small amounts ingested. Osterloh and associates calculated the lethal oleander leaf dose of their patient to be approximately 4 gm.[3] Practicing physicians should understand the potential lethal properties of oleander and its availability throughout the world.
